# A rare case of heteropaternal twin calves after natural mating in Brazil

**DOI:** 10.1590/1984-3143-AR2020-0217

**Published:** 2021-02-16

**Authors:** Fernanda Luiza Facioli, Gabriela da Fonseca Bezutti, Rodrigo Saraiva Bender, Mariana Groke Marques, Carlos Bondan, Eraldo Lourenso Zanella, Marcelo Bertolini, Ricardo Zanella

**Affiliations:** 1 Curso de Medicina Veterinária, Faculdade de Agronomia e Medicina Veterinária, Universidade de Passo Fundo – UPF, Passo Fundo, RS, Brasil; 2 Programa de Pós-graduação em Bioexperimentação, Curso de Medicina Veterinária, Faculdade de Agronomia e Medicina Veterinária, Universidade de Passo Fundo – UPF, Passo Fundo, RS, Brasil; 3 Embrapa Suínos e Aves, Concórdia, SC, Brasil; 4 Programa de Pós-graduação em Produção e Sanidade Animal, Instituto Federal Catarinense – IFC, Concórdia, SC, Brasil; 5 Programa de Pós-graduação em Agronomia, Curso de Medicina Veterinária, Faculdade de Agronomia e Medicina Veterinária, Universidade de Passo Fundo – UPF, Passo Fundo, RS, Brasil; 6 Faculdade de Veterinária, Universidade Federal do Rio Grande do Sul – UFRGS, Porto Alegre, RS, Brasil

**Keywords:** twin birth, beef cattle, heteropaternal superfecundation

## Abstract

Twin birth is a complex condition observed in most livestock animals, when the female gives birth to two or more offspring, generally out of the same mating. In cattle, it is a rare condition (3 to 5%) and depends on the genetic background and environmental factors. Twin birth is a result of multiple ovulations, being more common in dairy rather than in beef cattle. Calves could be monozygous or dizygous, with the same or of different sexes. When twins are born with different sexes, a sexual condition called Freemartinism occurs in between 90 to 97% of pregnancies, causing infertility in the female calf. Knowing that the twin rate is rare in commercial beef cattle, here we present an even rarer case of twin birth from two different sires after natural mating, also called heteropaternal superfecundation.

## Introduction

Reports on the bovine twin calving were first published in the early 1900s (Lillie, 1916). Decades later, an article published in *Science* suggested that fraternal twin calves often have two distinct blood groups, i.e., their own and that of their twin, and this was the base conceptual foundation of acquired immunological tolerance (Owen, 1945). Between 3 to 5% of all pregnancies in dairy cattle result in twins (Gáspárdy et al., 2018). However, the incidence of twins in beef cattle is less frequent generally not exceeding 1% in most beef herds (Esteves et al., 2012). Even rarer is the heteropaternal superfecundation (HS). It is a process characterized by the fertilization of two or more ova by different males during the same reproductive cycle, and are generally reported when phenotypically different characteristics (e.g. coat color patterns) are noted (McClure et al., 2017). So far, the rate of HS has not been fully studied. However, a retrospective study in Ireland reported a proportion of 0.98% of HS (0 to 2.65%) when 4902 births in cattle where evaluated both twins. Twins and the sire(s) were genotyped by SNP genotype testing (McClure et al., 2017)

In cattle, there is a great possibility to occur a common intersex condition called Freemartinism in dizygous twins born with different sexes due to anastomoses between the two placental vascular systems (Esteves et al., 2012). This condition affects the heifer twin by the underdevelopment of the female reproductive tract because of the Anti-Müllerian hormone (AMH) secretion by the male Sertoli cells that reach the female fetus (Lakshman and Kumar, 2019). The twin male calf can also be negatively affected, revealing reduced testicle size at times.

The objective of the present communication, therefore, was to describe male/female heteropaternal twin calves, confirmed by DNA parentage testing, born without clinical signs of freemartinism.

## Case presentation

This case report was followed during a theriogenology class of the veterinary course. It followed the principles set down by the animal care procedures of the Ethics Committee on Animal Utilization of the University of Passo Fundo CEUA-013/2019.

This is a case of an 8-years old crossbred cow [½ Chalores x (¼ Guzerá x ¼ Nelore)] bought as a rodeo cow by a cattle breeder when was 6 years old, the animal kept in Fazenda das Laranjeiras (Muitos Capões – RS – Brazil; 28°19'28.5”S 51°11'18.5”W). The farm has no history of twin births and records of the cow's previous pregnancy are unknown. The animals (n=120) of Muitos Capões farm are raised in a free-range system in native grassland during the summer. During the winter season, the animals were moved to a wheat and ryegrass pasture, receiving only mineral salt (containing only sodium chloride). The breeding season started on the 1st of September until the end of January (Spring-Summer), using natural service with two Guzerá and one Devon bulls. No hormonal protocols for reproductive synchronization is used on the farm. Animals received regular vaccination for foot-and-mouth disease, Brucellosis and Clostridiosis. No reproductive vaccination was conducted.

**Figure 1:**
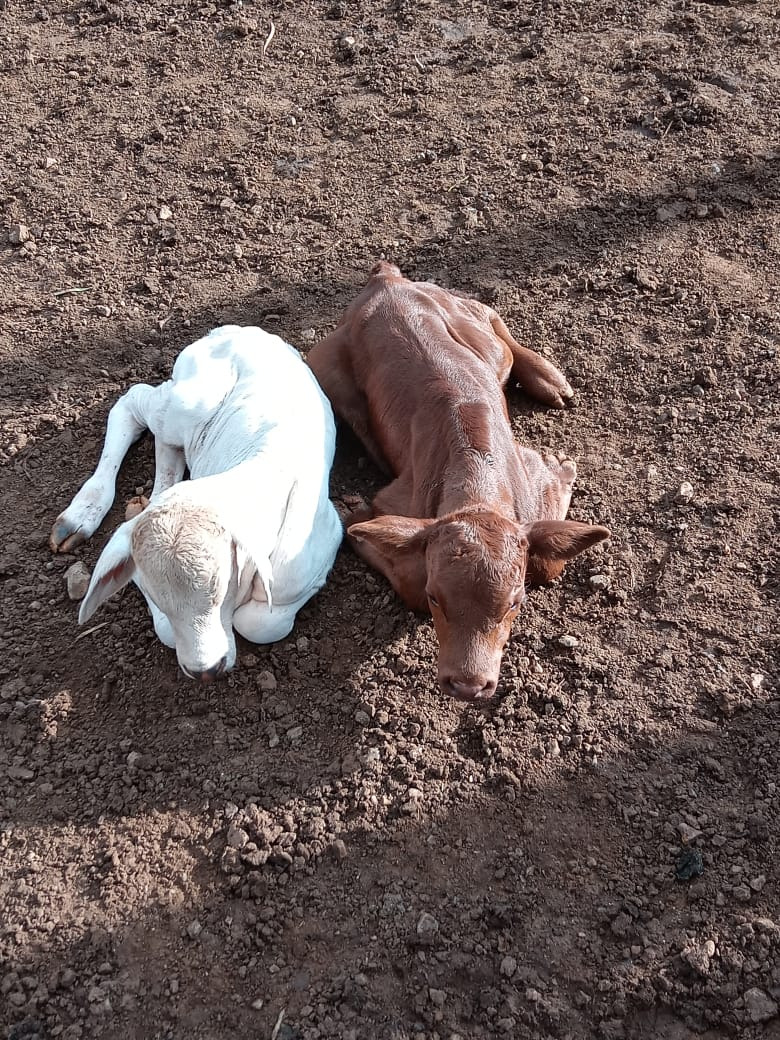
Calves with 3 days old presenting different phenotypes, a white female calf born of a Guzerá Bull, and the brown male calf born of a Devon Bull.

On June 18, 2020, the cow gave birth to twins of different sexes. Interestingly, calves had completely different phenotypes with different coat color patterns, which raised the attention of the cattle breeder (Figure 1). The male (ID: BD 01) was born with a brown coat color characteristic of the Devon bull; the female (ID: BG 02) presented a characteristic phenotype of Guzerá cattle. Hair samples from both calves, the cow, and the three bulls were collected to proceed with microsatellite parentage testing to identify the paternity of the twins at the VRGEN Laboratory (Araçatuba - SP - Brazil). Thirteen loci were analyzed according to the recommendations of the International Society for Animal Genetics (ISAG) (ISAG, 2008) tested by Van Eenennaam et al. (2007) and Vignal et al. (2002). Genotyping results confirmed that the two calves were born from the same cow, however from two different sires, the male calf from the Devon bull (ID: TD 03), and the female calf from one of the two Guzerá bulls (ID: TG 01), as presented in Annex 1. Further, the gynecological examination of the female calve was conducted as described by Almeida and Resende (2012). Interestingly, the female calve was not affected by the freemartin condition or any other visual anomaly using clinical examination.

## Discussion

Twin birth is a complex trait with multiple causal factors that include physiologic as well as genetic components (Johansson et al., 1974). It is a common condition observed in other species, such as sheep, pigs, goats, cats, and dogs (Berry et al., 2020)⁠. However, cattle are known to be a monotocous species, and in most of the cases, a successful pregnancy results in the birth of only one calf (Lu, 2014)⁠. Occasionally, the reproductive process in cattle can result in the birth of twins as a result of the genetic background, environmental factors, and their interactions (Johansson et al., 1974)⁠. Even knowing that the natural incidence of multiple births in cattle is very low, some variations on the twinning rate can be explained by breed differences and environmental factors, such as feeding and management systems, parity order, cow's age, the season of the year and a geographic location (Lu, 2014)⁠.

Based on the average of the twin rate found in the literature, assuming a prevalence of 1% of the twin rate, where we have a 50% chance to occur an intersex, and 5% of the chance for the female to be normal and 1% of different sires, it results in a probability of this case to be 1:2.5 millions. Here we have observed a twin birth from an 8-years old cow that was mainly kept in a native grassland with a high amount of legume forage (Red Clover). Unfortunately, previous information on the pregnancies of this cow is unknown. Although there were no studies about the percentage of twinning pregnancies in beef cattle resulting in calves born out of different sires, such condition commonly draws media attention. Some recent cases reported by the media include a Holstein Friesian cow that gave birth to twins from different sexes and phenotypically different, born of Hereford and Holstein Friesian bulls from the herd, produced also by natural mating, as our report (Irish Farm Centre, 2014)⁠. A similar case of heteropaternal superfecundation was reported by the media in 2016 when a Black Angus cow gave birth to twins with different sexes and physical characteristics from two different sires - one a cross with Charolais and the other across with black Simmental (Gannett Company, 2016)⁠. However, neither cases had a parentage genetic testing reported to confirm the paternity of the calves, speculating the different paternal origin only by the phenotype. We have used microsatellite testing to ascertain the parentage of the animals (Vignal et al., 2002)⁠. The use of microsatellite for parentage test is an exclusion assay. Therefore, we tested all possible bulls used in the breeding season, resulting in the high probability for the calves to be born out of different sires. The microsatellite profiles of the used bulls were unique for 11 markers, with an exception of two loci (TGLA126 and the ETH3), indicating the high polymorphic condition of the used markers.

## Conclusion

Our results have confirmed a rare case of heteropaternal superfecundation, which has produced two calves born from different bulls, with the female calf not being affected by freemartinism condition after clinical examination.

## Annex 1.

DNA parentage testing using 13 Microsatellites BD1 – Male Devon calf, BG2 – Female Guzerá calf, according to ISAG.
